# Endotrophin, an extracellular hormone, in combination with neoepitope markers of von Willebrand factor improves prediction of mortality in the ECLIPSE COPD cohort

**DOI:** 10.1186/s12931-020-01461-6

**Published:** 2020-07-30

**Authors:** Sarah R. Rønnow, Lasse L. Langholm, Morten A. Karsdal, Tina Manon-Jensen, Ruth Tal-Singer, Bruce E. Miller, Jørgen Vestbo, Diana J. Leeming, Jannie M. B. Sand

**Affiliations:** 1 Nordic Bioscience A/S, Herlev, Denmark; 2 COPD Foundation, Miami, Florida, USA; 3 R&D Respiratory Therapy Area Unit, GlaxoSmithKline, King Of Prussia, PA USA; 4 Division of Infection, Immunity and Respiratory Medicine, University of Manchester, Manchester, UK

**Keywords:** COPD, Biomarkers, Extracellular matrix

## Abstract

**Background:**

Lung epithelial damage, activation of the wound healing cascade, and remodeling of the extracellular matrix (ECM) play a major role in chronic obstructive pulmonary disease (COPD). The pro-peptide of type VI collagen has been identified as the hormone endotrophin. Endotrophin has been shown to promote fibrosis and inflammation, whereas von Willebrand factor (VWF) is a crucial part of wound healing initiation. Here, we assessed the released and activated form of VWF and endotrophin, the pro-peptide of type VI collagen, serologically to investigate their association with mortality in COPD subjects alone or in combination.

**Methods:**

One thousand COPD patients with 3 years of clinical follow-up from the Evaluation of COPD Longitudinally to Identify Predictive Surrogate Endpoints (ECLIPSE) cohort were included. Serum and heparin plasma were collected at 6 months and 1 year, respectively. Competitive ELISA utilizing specific monoclonal antibodies assessed endotrophin/type VI collagen formation (PRO-C6), VWF release (VWF-N), and activated VWF (VWF-A). Biomarker levels were dichotomized into high and low as defined by receiver operating characteristic (ROC) curves based on mortality data. Kaplan-Meier analysis was used to determine hazard ratios for all-cause mortality for biomarkers alone or in combination.

**Results:**

High levels of PRO-C6, VWF-A, and VWF-N have previously been shown to be individually associated with a higher risk of mortality with hazard ratios of 5.6 (95% CI 2.4–13.1), 3.7 (1.8–7.6), and 4.6 (2.2–9.6), respectively. The hazard ratios increased when combining the biomarkers: PRO-C6*VWFA 8.8 (2.8–27.7) and PRO-C6*VWFN 13.3 (5.6–32.0). Notably, PRO-C6*VWF-N increased more than 2-fold.

**Conclusion:**

We demonstrated that by combining two pathological relevant aspects of COPD, tissue remodeling, and wound healing, the predictive value of biomarkers for mortality increased notably.

## Introduction

Identification of chronic obstructive pulmonary disease (COPD) patients at risk is of high importance since it is the third leading cause of death worldwide [1]. COPD is characterized by chronic airway obstruction leading to chronic inflammation, tissue destruction, remodeling of the extracellular matrix (ECM), and small airway fibrosis [2–4]. Small airway fibrosis is believed to be caused by an altered repair response and activation of the wound healing cascade [4]. Combining biomarkers that reflect different aspects of the disease pathology could, therefore, provide us with a valuable tool to assess mortality risk in COPD patients.

Repeated exposure insults such as micro-particle pollution (cigarette smoke) cause lung injuries in COPD patients, thereby exposing the basement membrane and, eventually, the interstitial matrix [5]. In response to endothelial damage, von Willebrand factor (VWF) is released from the endothelia and activated which initiates the primary response/hemostasis by recruiting platelets that release platelet-derived growth factors (PDGF) and transforming growth factor-beta (TGF-β)-1. These growth factors stimulate endothelial cell-regeneration and the production of new ECM proteins by fibroblasts in order to repair the underlying damaged connective tissue [6–8]. In particular, Type VI collagen which consists of domains that are highly homologous to the ones found in VWF, has been shown to play a role in the activation of platelets due to its ability to bind to both VWF and platelets [9, 10]. The type VI collagen α3 chain is one of the most abundant collagens in the adult murine lung thus it is of interest to investigate its role in COPD [11]. Moreover, type VI collagen C5 domain of the α3 chain, endotrophin, is a signaling fragment that is released during the remodeling of the ECM [12]. Endotrophin stimulates fibrosis, activates endothelial cell migration, and promotes macrophage infiltration to damaged tissue [12, 13]. Endotrophin has also been shown to stimulate the production of TGF-β [14], suggesting a crucial role in the development and sustainment of fibrosis, possibly in COPD pathogenesis.

We hypothesized that combining biomarkers of two different pathology aspects of COPD, such as ECM remodeling and wound healing, would increase prognostic accuracy and thereby aid in the identification of COPD subjects at higher risk of mortality outcome. We assessed a biomarker of the profibrotic hormone endotrophin (PRO-C6) and biomarkers reflecting newly released and actived VWF. Activated VWF was assessed by targeting the cleavage-site for the metalloproteinase ADAMTS13 exposed by unfolding during activation (VWF-A), whereas VWF formation/release was evaluated by targeting the released pro-peptide (VWF-N).

## Materials and methods

### Study design and participants

The study design of Evaluation of COPD Longitudinally to Identify Predictive Surrogate End-points (ECLIPSE) (clinicaltrials.gov identifier NCT00292552; GSK study code SCO104960) has been fully described previously [15]. The current analysis was based on a three-year clinical follow-up and performed on a subpopulation of the full ECLIPSE study of 1000 COPD consisting of the 500 patients progressing the most and the 500 patients progressing the least as defined as FEV_1_ decline. For the present post hoc analysis, we used clinical and biomarker data from 898 patients obtained at month six, year one, and year three. The study complied with the declaration of Helsinki and good clinical practice guidelines and was approved by the relevant ethics and review boards. All participants provided informed consent.

### Quantification of serological biomarkers

Serum and heparin plasma samples were prepared from participants in the fasting state and stored at − 80 °C until analyzed. PRO-C6 was measured in month six serum samples while VWF-A and VWF-N were measured in year one plasma samples. Competitive ELISAs, utilizing monoclonal antibodies recognizing specific neo-epitopes, were used to assess endotrophin/type VI collagen formation (PRO-C6), VWF formation/ endothelial release (VWF-N), and activated VWF (VWF-A) [16, 17] (Nordic Bioscience A/S, Herlev, Denmark). Measurements were performed previously in a blinded manner, according to the manufacturer’s instructions [18, 19].

### Statistics

Receiver operating characteristic (ROC) curve analysis was used to dichotomized data into high versus low based on a cut-off from Youden Index criterion based on mortality data. Kaplan-Meier survival curves compared the mortality risk for patients belonging to the high or the low group of biomarker levels. Hazard ratios were extracted from the Kaplan-Meier analysis. All tests were performed in the Statistical Software MedCalc version 14.8.1 (MedCalc Software bvba, Ostend, Belgium). A *p*-value < 0.05 was considered statistically significant.

## Results

### Basic demographics

Baseline characteristics for survivors and deceased are listed in Table 1. The survivors are significantly younger, have a lower modified medical research council (mMRC) score, more are currently smoking, but their smoking pack-years history is lower.

**Table 1:** Basic demographics

Subset of the ECLIPSE cohort	Survivors	Deceased	*P*-values
**n**	871	27	
**Sex, M/F [n (%)]**	555 (64%) / 316 (36%)	17 (63%) / 10 (37%)	*P* = 0.9358 #
**Age, years**	62.8 (62.4–63.3)	68.6 (66.6–70.5)	*P* < 0.0001 £
**BMI, kg/m**^**2**^	26.8 (26.4–27.2)	26.6 (23.8–29.4)	*P* = 0.8837 £
**FEV**_**1**_**, L**	1.30 (1.27–1.33)	1.81 (1.04–1.33)	*P* = 0.2068 £
**FEV**_**1**_**% predicted**	46.5 (45.6–47.5)	45.4 (40.9–50.0)	*P* = 0.6986 £
**Current smokers [n (%)]**	330 (38%)	3 (11%)	*P* = 0.0046 #
**Smoking history, pack-years**	46.7 (45.0–48.3)	59.0 (41.7–76.3)	*P* = 0.0152 £
**mMRC [IQR]**	1 (1–2)	2 (1–3)	*P* = 0.0486 #
**GOLD stage [IQR]**	2 (2–3)	2 (2–3)	*P* = 0.5904 #

£ T-test and # Chi-squared test. Data are shown as median (95% CI) unless stated otherwise. FEV_1_, forced expiratory volume in 1 second (post-bronchodilator); mMRC, modified medical research council (dyspnea scale); GOLD, Global initiative for chronic Obstructive Lung Disease.

### Combining PRO-C6 with VWF-A and VWF-N improves prediction of mortality

**Fig. 1:**
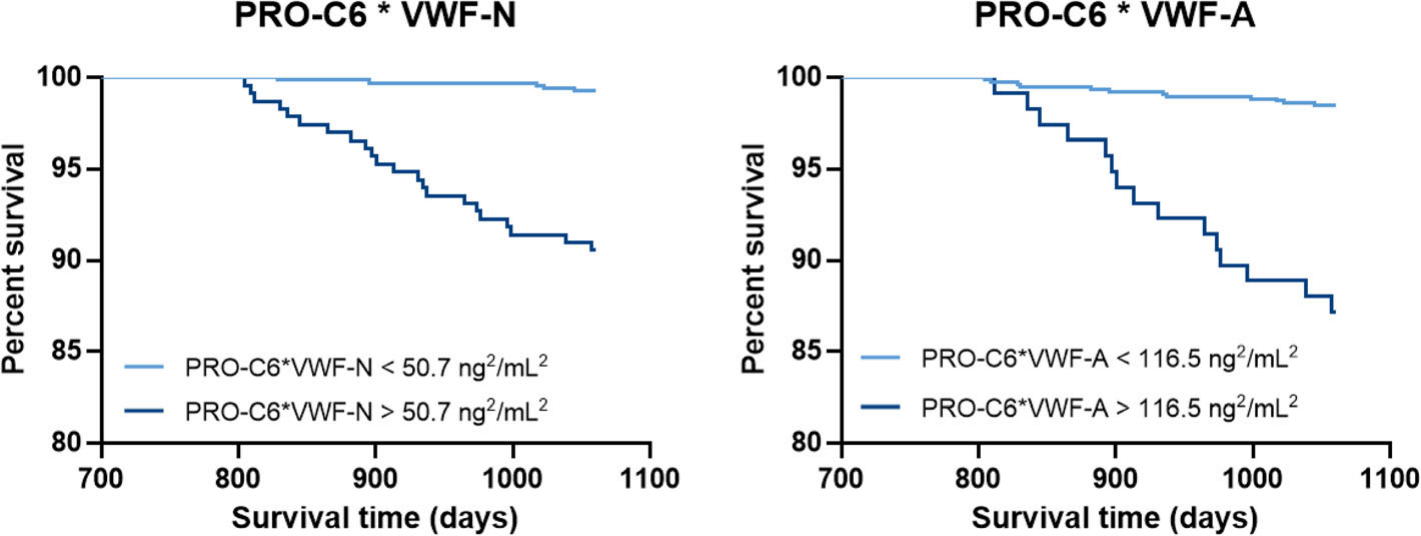
Kaplan-Meier survival curves for high versus low biomarker levels using a cut-off of 50.7ng^2^/mL^2^ for PRO-C6*VWF-N and 116.5ng^2^/mL^2^ for PRO-C6*VWF-A. Individuals with high biomarker levels of PRO-C6*VWF-N and PRO-C6*VWF-A showed a higher number of deaths (*n* = 22 and *n* = 15) within the study period compared to subjects with low biomarker levels of PRO-C6*VWF-N and PRO-C6*VWF-A (*n* = 5 and *n* = 12)

**Fig. 2:**
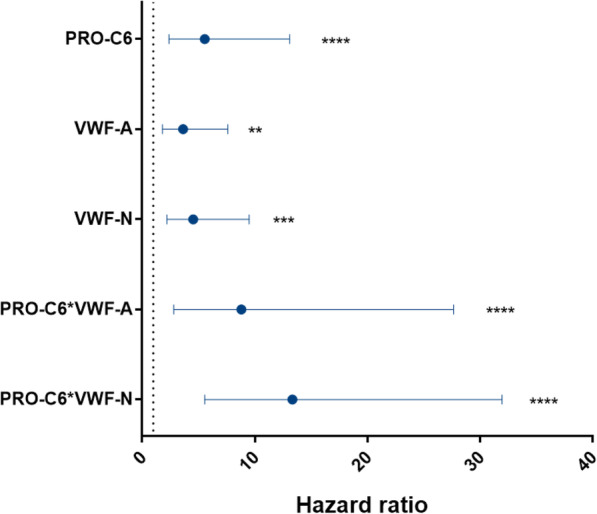
Hazard ratios for all-cause mortality were extracted from the Kaplan-Meier analysis and presented as mean ± 95% CI. Asterisks indicate statistical significance**p < 0.01; ***p < 0.001; ****p < 0.0001

PRO-C6, VWF-A, and VWF-N have already been shown to be independent predictors of all-cause mortality [18, 19]. To investigate if PRO-C6 combined with biomarkers of VWF processing could provide additional prognostic value, we multiplied the biomarkers. Data were dichotomized by a ROC analysis based on mortality data. The ROC curve for PRO-C6 multiplied by VWF-A (PRO-C6*VWF-A) had an area under the curve (AUC) of 0.735 (*p* < 0.0001) and provided a cut-off 116.5ng^2^/mL^2^ for the identification of participants who died during the total follow-up time with a sensitivity of 55.6 and a specificity of 88.3 (data not shown). The ROC for PRO-C6 multiplied with VWF-N (PRO-C6*VWF-N) had an AUC of 0.788 (*p* < 0.0001) with the cut-off 50.7 ng^2^/mL^2^ for identification of subjects who died. This curve had a sensitivity of 81.5 and specificity of 76 (data not shown). Based on the ROC analyses, data were dichotomized and analyzed by the Kaplan-Meier survival curves (Fig. 1). Kaplan-Meier survival analysis and hazard ratios evaluated the prognostic value of biomarkers alone or in combination for all-cause mortality after 3 years. Previously it was shown that high levels of PRO-C6, VWF-A, and VWF-N were associated with a higher risk of mortality with hazard ratios of 5.6 (95% CI 2.4–13.1), 4.6 (95% CI 2.2–9.6) and 3.7 (95% CI 1.8–7.6), respectively [18, 19]. These values were significantly increased when combining PRO-C6 with the VWF processing biomarkers VWF-A 8.8 (2.8–27.7) or VWF-N to hazard ratios of 8.8 (95% CI 2.8–27.7) and 13.3 (95% CI 5.6–32.0), respectively. The hazard ratios are displayed in Fig. 2 for the single biomarkers and composite biomarkers. All hazard ratios were tested for cofounders and were found to be independent predictors of all-cause mortality (data not shown).

## Discussion

It is of high importance to identify COPD patients at risk of rapid progression and mortality. Currently, plasma fibrinogen is the only Food and Drug Administration (FDA) qualified prognostic biomarker for all-cause mortality in COPD based on data from the ECLIPSE cohort [20]. However, fibrinogen only reflects chronic wound healing but not fibrosis. Since COPD is a very heterogeneous disease, it is essential to investigate more of the pathological features of COPD at the same time. In the present manuscript, post hoc analysis revealed that combining different aspects of the disease, the biomarkers of pro-fibrotic endotrophin (PRO-C6) and VWF processing, further improved the prediction of all-cause mortality in COPD subjects.

ECM remodeling leads to the release of ECM fragments, also referred to as neo-epitopes, into the circulation, and these protein fragments have been proposed as disease activity biomarkers. Neo-epitopes may be viewed as end-products of repair processes in the lungs [21]. ECM remodeling, including both formation and degradation of the lung tissue, has been shown to be increased in patients with COPD [22–25]. Furthermore, an accelerated rate of remodeling was associated with acute exacerbations of COPD and an elevated risk of mortality [18, 22–26]. Abdillahi et al. showed that type VI collagen mRNA and protein are increased in the lungs of COPD patients when compared to controls [27]. Stolz et al. found PRO-C6 to be associated with lung function and survival, while Bihlet et al. found PRO-C6 to be correlated with blood eosinophils and lung function [22, 28]. Endotrophin, a fragment of type VI collagen, has been shown to modulate cell-cell interactions, stimulate proliferation of mesenchymal cells, and prevent cell apoptosis [29, 30]. These data all indicate the importance of type VI collagen in the development of fibrosis and that collagen type VI may play an essential role in the pathogenesis of COPD as well.

**Fig. 3:**
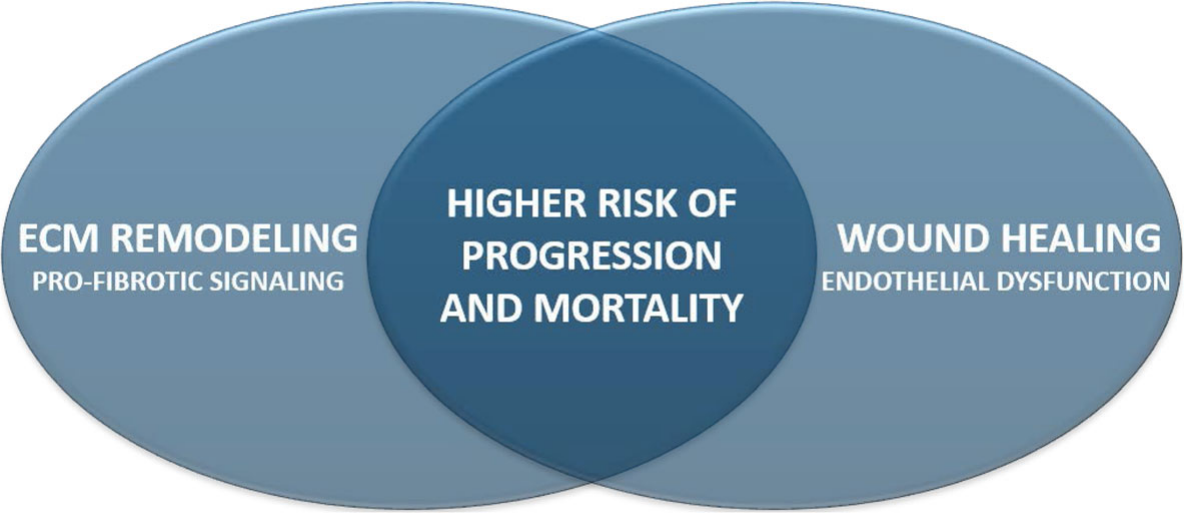
The combination of high ECM remodeling and wound healing increases the risk of progression and mortality

Previously, we showed that VWF-A relates to a more acute state of COPD during exacerbations, whereas VWF-N relates to the more chronic state of COPD emphysema, that VWF could potentially be utilized as a marker for endothelial dysfunction and inflammation [19], which have also previously been suggested [6, 31]. In the present study, we demonstrate that the highest hazard ratio was obtained by combining the measurement of PRO-C6 and VWF-N, which could reflect that the patients suffering from a more advanced profibrotic state and emphysema are also the ones at risk for poor outcomes. To understand this increased prognostic accuracy, we realize that wound healing is a continuous process in many tissues, and PRO-C6, which is produced by fibroblasts, may be expressed in multiple organs, such as skin [32]. Thus, as illustrated in Fig. 3, the combination of two processes related to COPD may better capture the activity in the lungs, and patients who have both wound healing and activated fibroblast are more likely to progress and have a fatal outcome. This was in concordance with the findings by Hurana et al., showing that emphysematous changes assessed by CT scan were able to predict mortality in COPD patients [33].

Limitations of this study include the low number of deceased COPD participants in the subpopulation of the ECLIPSE study. Furthermore, PRO-C6 was measured in serum at month six, whereas VWF-A and VWF-N were measured at year 1 in heparin plasma, which posed difficulties when comparing and interpreting the results. Therefore, these data need to be confirmed in a larger longitudinal cohort, which assessed mortality as an outcome measure.

## Conclusion

We demonstrate in this study that combining biomarkers that reflect two different aspects of tissue repair in COPD is of importance. Combining the profibrotic hormone endotrophin, also capturing type VI collagen formation, and wound healing processing improved the prediction of all-cause mortality in COPD. Increased wound healing and endotrophin levels may indicate an over-active repair process and fibrosis, which may explain why these biomarkers are associated with mortality in COPD.

## Data Availability

The datasets used and/or analyzed during the current study are available from the corresponding author on reasonable request.

## Abbreviations

AUC: Area under the curve
COPD: Chronic obstructive pulmonary disease
ECLIPSE: Evaluation of COPD Longitudinally to Identify Predictive Surrogate Endpoints
ECM: Extracellular matrix
FDA: Food and Drug Administration
FEV_1_: Forced expiratory volume in one second
GOLD: Global initiative for chronic Obstructive Lung Disease.
mMRC: Modified medical research council
PDGF: Platelet-derived growth factors
PRO-C6: Endotrophin/type VI collagen formation
ROC: Receiver operating characteristic
TGF-β: Transforming growth factor-beta
VWF von: Willebrand factor
VWF-A: Activated VWF
VWF-N: VWF release
